# Serum Metabolomics Study to Screen Potential Biomarkers of Lung Cancer Risk in High Natural Background Radiation Areas of Thailand: A Pilot Study

**DOI:** 10.3390/cancers16244182

**Published:** 2024-12-15

**Authors:** Narongchai Autsavapromporn, Aphidet Duangya, Pitchayaponne Klunklin, Imjai Chitapanarux, Chutima Kranrod, Churdsak Jaikang, Tawachai Monum, Atchara Paemanee, Shinji Tokonami

**Affiliations:** 1 Division of Radiation Oncology, Department of Radiology, Faculty of Medicine, Chiang Mai University, Chiang Mai 50200, Thailand; aphidet.d@cmu.ac.th (A.D.); pitchayaponne.kl@cmu.ac.th (P.K.); imjai.chitapanarux@cmu.ac.th (I.C.); 2 Institute of Radiation Emergency Medicine, Hirosaki University, Hirosaki 036-8564, Aomori, Japan; kranrodc@hirosaki-u.ac.jp (C.K.); tokonami@hirosaki-u.ac.jp (S.T.); 3 Toxicology Section, Department of Forensic Medicine, Faculty of Medicine, Chiang Mai University, Chiang Mai 50200, Thailand; churdsak.j@cmu.ac.th (C.J.); tawachai.m@cmu.ac.th (T.M.); 4 National Omics Center, National Center for Genetic Engineering and Biotechnology (BIOTEC), National Science and Technology Development Agency (NSTDA), Khlong Luang, Pathum Thani 12120, Thailand; atchara.pae@biotec.or.th; 5 Food Biotechnology Research Team, Functional Ingredients and Food Innovation Research Group, National Center for Genetic Engineering and Biotechnology (BIOTEC), National Science and Technology Development Agency (NSTDA), Khlong Luang, Pathum Thani 12120, Thailand

**Keywords:** biomarkers, D-sphingosine, indoor radon, lung cancer, metabolomics, UHPLC-HRMS

## Abstract

**Background:** Indoor radon is a significant risk factor for the development of LC. This study aimed to identify potential biomarkers for LC risk in high background radiation areas using a metabolomics approach (UHPLC-HRMS). **Methods:** Based on the indoor radon activity concentration measurements in the Kong Khaek subdistrict, serum samples were collected from 45 nonsmoker or former smoker participants, comprising 15 LC patients and 30 matched healthy controls (low- and high-radon groups, respectively). **Results:** A total of 90 and 111 differential metabolites were identified in the LC group compared with the low- and high-radon groups, respectively, using criteria such as a variable importance in projection (VIP) of >1, a fold change (FC) of >1 or <0.5, and a *p* value of <0.05. Receiver operating characteristic (ROC) curves (an AUC of ≥ 0.9) indicated that 30 and 21 of these metabolites had the potential to serve as biomarkers of LC development in the low- and high-radon groups, respectively. The KEGG pathway enrichment analysis suggested that D-sphingosine may have been a candidate biomarker associated with LC in both groups. **Conclusions:** Overall, this study provides new insights into metabolic biomarkers for screening LC development in high-risk individuals with prolonged exposure to indoor radon. Further large-scale studies are needed to validate our results.

## Simple Summary

Lung cancer (LC) is the leading cause of cancer-related mortality in Thailand, particularly in the Chiang Mai province. Beyond cigarette smoking, long-term exposure to indoor radon is a significant risk factor for the development of LC. In Kong Khaek, a subdistrict of the Mae Chaem district in Chiang Mai, the average indoor radon activity concentration exceeds both global and national averages. Understanding the impact of chronic low-dose radiation exposure on human populations living in high-background radiation areas is crucial. However, no specific biomarkers are currently available to detect LC resulting from prolonged exposure to indoor radon.

## 1. Introduction

Lung cancer (LC) is the most common malignant tumor worldwide, with the highest incidence and mortality rates [1]. Approximately 85% of all LC patients have non-small cell LC (NSCLC), which includes squamous cell carcinoma, large cell carcinoma, and lung adenocarcinoma, while the remaining 15% have small cell LC (SCLC). Current LC treatment options include surgery resection, chemotherapy, radiotherapy, immunotherapy, targeted therapy, and combination treatments. Due to the lack of typical symptoms and poor prognoses, the 5-year survival rates for NSCLC and SCLC are only 26% and 7%, respectively [1]. Common diagnostic methods for LC primarily rely on chest X rays, magnetic resonance imaging (MRI), computed tomography (CT), and positron emission computed tomography (PET) [2]. Over 70% of LC patients are diagnosed at advanced stages due to the absence of biomarkers for screening high-risk individuals [2,3]. Therefore, early detection is critical for improving LC prevention, management, and prognosis. In Thailand, LC is the second most common cancer in men and the fifth most common in women. According to the World Health Organization (WHO), LC is the leading cause of cancer-related mortality in upper northern Thailand (UNT), particularly in the Chiang Mai province, compared to other regions [4]. Major LC risk factors in Chiang Mai include cigarette smoking, air pollution, chronic lung disease, lifestyle factors, and radon (^222^Rn) exposure. While cigarette smoking remains the primary LC risk factor in Chiang Mai, indoor radon is the second leading risk factor and the main risk factor for nonsmokers [5,6].

Radon and its short-lived decay products are classified as human carcinogenic factors (group 1) by the International Agency for Research and Cancer (IARC) [7,8]. Radon is a naturally occurring radioactive noble gas that emits high linear energy transfer (LET) α particles as part of the decay chains of its uranium series (^238^U). It is commonly found in rocks, soil, ground water, building materials, and air. With a half-life of 3.82 days, radon is odorless, colorless, and invisible. The increased risk of LC development from indoor radon exposure and its decay products is due to α particles irradiating the bronchial epithelial cells [8,9,10,11]. Epidemiological studies suggest that the latency period for LC development due to indoor radon exposure ranges from 5 to 25 years [9]. According to the World Health Organization (WHO), long-term exposure to indoor radon is estimated to increase LC risk by 16% per 100 Becquerel (Bq)/m^3^ of measured indoor radon activity concentration in dwellings [12,13]. The WHO recommends an action level of 100 Bq/m^3^ for indoor radon activity concentrations in residential settings [12]. Chiang Mai has significantly higher natural background radiation than other regions in Thailand due to its elevated levels of indoor radon and its decay products [14,15,16]. Our previous study demonstrated that the average indoor radon activity concentration in Chiang Mai exceeded both the national and global averages of 16 and 39 Bq/m^3^, respectively [14,15,16]. Additionally, the indoor radon activity concentrations in Chiang Mai are higher during the dry season due to burning compared to the non-burning season [15,16]. These findings highlight the need to raise awareness about the long-term health effects of indoor radon among populations in the high natural background radiation areas of Chiang Mai.

A lack of early-stage LC detection can lead to poor diagnostic and treatment outcomes. To date, metabolites in human tissues and biofluids serve as novel biomarkers for the diagnosis, prediction, progression, and prognosis of LC. Metabolomics, characterized by its high selectivity and specificity, is an ideal tool for the noninvasive diagnosis and monitoring of LC. Metabolomics studies are conducted using nuclear magnetic resonance (NMR), high-performance liquid chromatography (HPLC), liquid chromatography mass spectrometry (LC-MS), gas chromatography mass spectrometry (GC-MS), and ultra-high-performance liquid chromatography coupled with high-resolution mass spectrometry (UHPLC-HRMS) to detect small molecular metabolites [17,18]. To the best of our knowledge, no metabolic studies have investigated novel biomarkers for LC in areas with high natural background radiation. The aim of this study was to determine the serum metabolomics profiles of LC patients and match healthy controls from both low- and high-radon groups using the UHPLC-HRMS technique. All participants were either nonsmokers or former smokers. Differences in metabolomic profiles among the three groups were analyzed using principal components analysis (PCA), partial least squares discriminant analysis (PLS-DA), and receiver operating characteristic (ROC) curve analysis, enabling the identification of potential biomarkers for LC. This study provides new insights into potential metabolic markers for LC screening in high-risk individuals exposed to chronic low-dose indoor radon.

## 2. Materials and Methods

### 2.1. Study Area

Kong Khaek is a subdistrict of the Mae Chaem district in Chiang Mai, located in UNT. It is surrounded by granite highland mountains and forested landscapes. The subdistrict comprises 12 villages, with a population of 6572 people across 2304 dwellings as of 2023. Between September 2022 and March 2023, indoor radon activity concentrations were measured using a passive radon-thoron discriminative monitor (RADUET) equipped with a solid-state track detector (CR-39). The estimated indoor radon activity concentration was approximately 18.5 ± 119 Bq/m^3^, with an average value of 40.8 ± 22.6 Bq/m^3^ [16]. Based on these measurements, we categorized the indoor radon activity concentrations into the following three groups according to the dwellings’ locations: low (<30 Bq/m^3^), moderate (30–50 Bq/m^3^) and high (>50 Bq/m^3^).

### 2.2. Partcipants

The transitional study was conducted on a selected group of residents of Kong Khaek who had lived in the study area for at least 15 years. We recruited a total of 135 participants (aged 18–93 years), including 50 LC patients and 85 healthy controls (HC), for this study. The HC group was divided into low- and high-radon subgroups based on their indoor radon activity concentration results. There were no restrictions on histology, gender, or cancer stage. Between September 2022 and August 2023, NSCLC patients were enrolled from the Division of Radiation Oncology, Department of Radiology, Maharaj Nakorn Chiang Mai University Hospital.

The inclusion criteria for both groups were as follows: age of >18 years old, no prior radiotherapy, and being either a nonsmoker or former smoker (more than 20 years since quitting) at the time of the interviews, which were conducted by trained researchers. Exclusion criteria for both groups included the following: not meeting the inclusion criteria, pregnancy, and having a history of other cancers. For the study, we randomly selected the LC and HC groups with an age range of between 30 and 80 years old (Table 1). All participants were informed about the purpose of this study, provided written consent, and completed a questionnaire prior to the collection of blood samples. The questionnaire covered topics such as smoking history, alcohol consumption, air pollution, diet, family history of cancer, and working history.

### 2.3. Sample Collection

Fasting blood samples (10 mL) were collected in coagulation-promoting tubes. The samples were incubated at room temperature for 30 min and then centrifuged at 4 °C and 3000× *g* for 10 min. The serum samples were taken and stored at −80 °C for further analysis.

### 2.4. Sample Preparation

After thawing in an ice-water bath, the serum samples (250 μL) were diluted with 250 μL of 100 mg/L 4-Chloro-L-phenylalanine dissolved in 70% methanol from Sigma-Aldrich (St. Louis, MO, USA). The mixture was vortexed for 30 s and subsequently centrifuged at 4 °C and 13,000× *g* for 10 min. The supernatant was transferred to a clean tube (1.5 mL) and dried under a vacuum at room temperature. Finally, the dried samples were redissolved by adding 500 μL of 10% (*v*/*v*) acetonitrile and then filtered through a hydrophilized polytetrafluoroethylene (HPTFE) membrane with a pore size of 0.22 μm for further UHPLC-HRMS analysis.

### 2.5. Ultra-High-Performance Liquid Chromatography Coupled with High-Resolution Mass Spectrometry (UHPLC-HRMS)

The UHPLC-HRMS analysis was performed using a UHPLC system (Vanquish; Thermo Fisher Scientific, Inc., Waltham, MA, USA) coupled with a Q Exactive^TM^ HF–X Quadruple—Orbitrap mass spectrometer system (Thermo Fisher Scientific, Inc., Waltham, MA, USA). The serum samples were separated on a Hypersil GOLD^TM^ Vanquish C18 column (1.9 μm, 2.1 × 100 mm, Thermo Fisher Scientific, Inc., Waltham, MA, USA) with a guard column and maintained at 30 °C and a flow rate of 0.3 mL/min. The mobile phase consisted of 0.1% (*v*/*v*) formic acid in water (phase A) and 0.1% (*v*/*v*) formic acid in acetonitrile (phase B). The injection volume was 2 μL. The gradient elution was follows: 0–1 min, 2% B; 1–18 min, 2% to 100% B; 18–20 min, 100% B; and 20–25 min, 100% to 2% B. The total run time was 25 min.

The MS detection was performed using a Q-Exactive^TM^ HF–X Orbitrap mass spectrometer with a heated electrospray ionization source (HESI) ion source (Waltham, MA, USA). Positive ions were detected using the full-scan MS1/data-dependent MS2 (dd-MS2) mode with the following specific parameters: full-scan MS1 resolution, 120,000; dd-MS2 resolution, 30,000; mass range, 70 to 1000 *m*/*z*; auxiliary gas heater temperature, 320 °C; capillary temperature, 320 °C; maximum injection time, 100ms; automatic gain control target, 3 × 10^6^; stepped N(CE) at 10, 20, and 40 eV; auxiliary gas, 10 arbitrary units (AU); sheath gas, 45 AU; sweep gas, 5 AU; and spray voltage, either 2.5 kV (negative) or 3.5 kV (positive) [19].

### 2.6. Data Processing and Statistical Analysis

The raw data processing was performed using Compound Discover 3.3 software (Thermo Fisher Scientific, Inc.). Normalization was carried out using quality control (QC) samples to identify the differential metabolites. The MS data were searched against the Human Metabolome Databases (HMDB) for metabolite annotation, including mzVault, mzCloud, and ChemSpider.

All data are presented as means ± standard deviations (SDs). Statistical analysis of the UHPLC-HRMS data was performed using MetaboAnalyst 6.0. Differences between the groups were analyzed using the Student’s *t*-test (for 2 groups) or ANOVA (for more than 2 groups). A *p* value of less than 0.05 was considered statistically significant. Differences in the serum metabolics among the three groups (LC and low- and high-radon) were explored using a principal component analysis (PCA) and a partial least squares-discriminate analysis (PLS-DA). The quality of the PCA and PLS-DA models was assessed using the values of Q^2^ (predictive parameter) and R^2^ (explanatory parameter). The selection criteria for the differential metabolites between the groups were a fold change (FC) of >1 or <0.5, a variable importance in the projection (VIP) of >1, and a *p* value of <0.05. A receiver operating characteristic (ROC) analysis was performed using MetaboAnalyst 6.0 to identify the potential LC biomarkers. A biomarker was considered to have high diagnostic value when the area under the curve (AUC) was ≥0.9. Additionally, the metabolic pathways and metabolite set enrichment analysis were conducted based on the Kyoto Encyclopedia of Genes and Genomes (KEGG) database.

## 3. Results

### 3.1. Study Population

This study included 15 NSCLC patients and 30 individuals in the HC group (Table 1). The HC group was evenly divided into 15 participants from the low-radon group and 15 from the high-radon group. All participants were either nonsmokers or former smokers. The mean ages of the participants were 62 ± 13.3, 61.1 ± 11.2, and 61.3 ± 13 years for the LC and low- and high-radon groups, respectively. No significant differences were observed between the ages and genders of the three groups.

### 3.2. Metabolic Proflie Analysis

**Figure 1:**
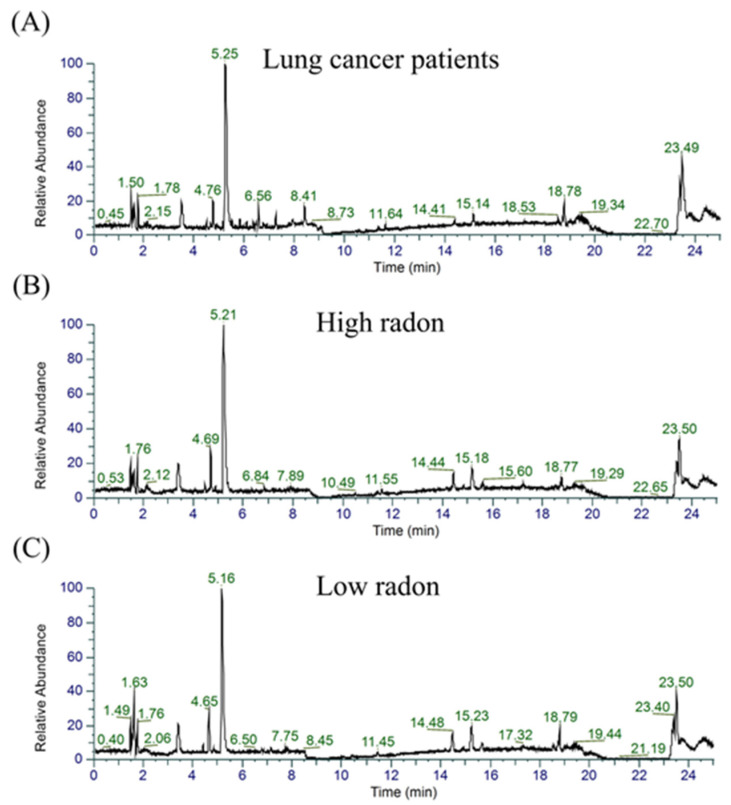
The base peak intensity chromatograms of the serum samples. The (**A**) LC, (**B**) high-radon, and (**C**) low-radon groups.

The UHPLC-HRMS technique was employed to perform the metabolomics analysis on the serum samples of the NSCLC patients and the HC groups (low- and high-radon). The base peak intensity chromatograms of the serum samples of the LC and high- and low-radon groups are presented in Figure 1. As expected, the base peak intensity varied between the three groups, with significant differences observed between 1 and 16 min.

### 3.3. Multivariate Data Analysis for the UHPLC-HRMS

**Figure 2:**
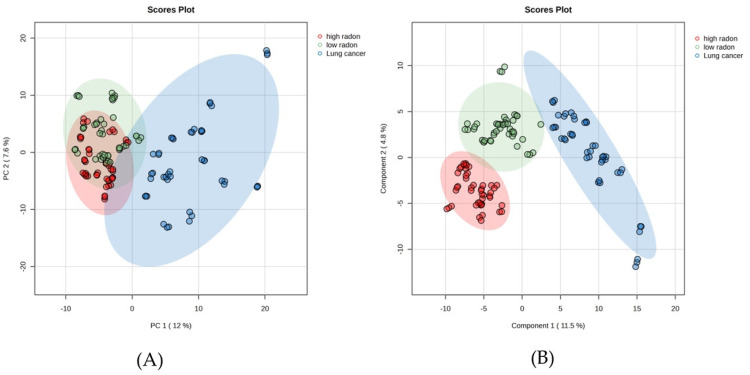
PCA and PLS-DA score plots for the LC and high- and low-radon groups. The (**A**) PCA and (**B**) PLS-DA models.

The PCA and PLS-DA models were used to characterize the differences in the metabolites among the LC and low- and high-radon groups (Figure 2). The PCA model’s results indicated that it was unable to distinguish differences in the serum metabolites among the three groups (Figure 2A). In contrast, the PLS-DA model demonstrated good discrimination among all groups (Figure 2B). The evaluation parameters for the PLS-DA model (Q^2^ = 0.98, R^2^ = 0.99) confirmed the accuracy and reliability of the metabolite analysis, as both values exceeded 0.9. Thus, the PLS-DA model was suitable for further screening of the differentially expressed metabolites.

### 3.4. Identification of the Differential Metabolites

#### 3.4.1. Identification of the Differential Metabolites Distinguishing the LC and Low-Radon Groups

**Figure 3:**
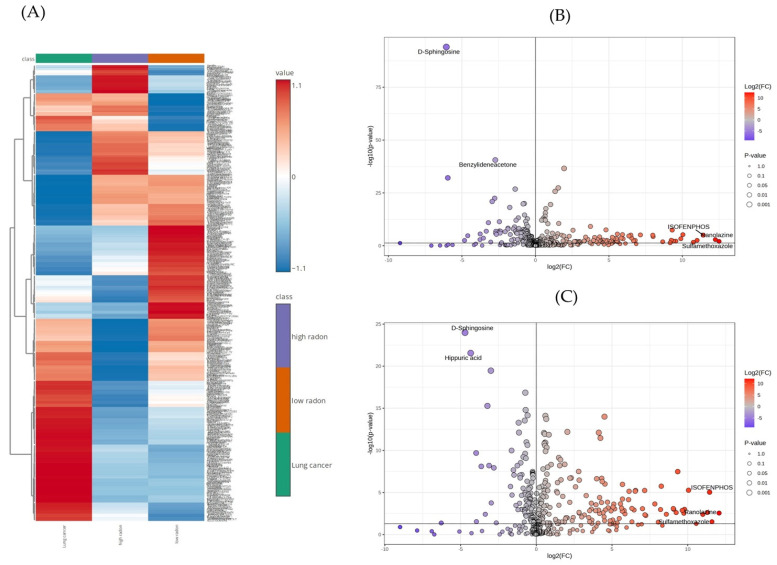
(**A**) Heatmap of the differential metabolites between the LC and low- and high-radon groups. A volcano plot of the metabolomics for the LC patients and the low-radon group (**B**) and the high-radon group (**C**).

Using the UHPLC-HRMS technique, a total of 449 differential metabolites were identified among all groups from the serum samples. As expected, some metabolites exhibited variations across the three groups (Figure 3A). To elucidate the differences in the serum metabolomics profiles between the LC and low- and high-radon groups, we first analyzed the metabolites between the LC and low-radon groups. A total of 263 metabolites were identified between the LC and low-radon group, of which 132 were up-regulated and 131 were down-regulated (Figure 3B). Based on the selection criteria (VIP of >1, FC of >1 or <0.5, and *p* of <0.05), 90 differential metabolite screening results were identified, with 54 upregulated and 36 downregulated metabolites in the LC group compared to the low-radon group (Supplemental Table S1).

The differential metabolites of the LC patients and the low-radon group were evaluated as potential biomarkers for LC development using a ROC analysis, which demonstrated high diagnostic power (AUC of ≥0.9). Differentiation between the LC patients and the low-radon group could be achieved by combining 30 potential metabolic biomarkers, of which 10 were downregulated and 20 were upregulated (Table 2). As shown in Table 2, D-sphingosine, benzylideneacetone, 2-[(5E,8E)-5,8-Tetradecadien-1-yl]cyclobutanone, and hydrocortisone succinate had AUC values of 1.00, along with high specificity and sensitivity (>0.98). These metabolites may be particularly useful for identifying LC patients and distinguishing them from individuals like those of the low-radon group.

#### 3.4.2. Identification of the Differential Metabolites Distinguishing the LC and High-Radon Groups

A total of 274 metabolites were identified between the LC and high-radon groups, of which 165 were up-regulated and 109 were down-regulated (Figure 3C). Based on the selection criteria (VIP of >1, FC of >1 or <0.5, and *p* of <0.05), 111 differential metabolites were identified, with 89 upregulated and 22 downregulated metabolites in the LC group compared to the high-radon group (Supplemental Table S2). Next, these differential metabolites were evaluated as candidate biomarkers for LC development using a ROC analysis (AUC of ≥0.9). Differentiation between the LC and high-radon groups could be achieved by combining 21 metabolites, comprising 14 upregulated and 7 downregulated metabolites (Table 3). As shown in Table 3, the AUC values for pirenzepine, ?-aspartylphenylalanine, D-sphingosine, nornorcapsaicin, hydrocortisone succinate, hippuric acid, aspartame, and (2E)-decenoic acid were 0.98, 0.97, 0.96, 0.96, 0.96, 0.95, 0.95, and 0.95, respectively, with high specificity and sensitivity (>0.87). These findings suggest their potential for identifying LC patients and distinguishing them from individuals similar to those in the high-radon group.

### 3.5. Pathway Analysis of the Differential Metoabolites Using the KEGG Database

**Figure 4:**
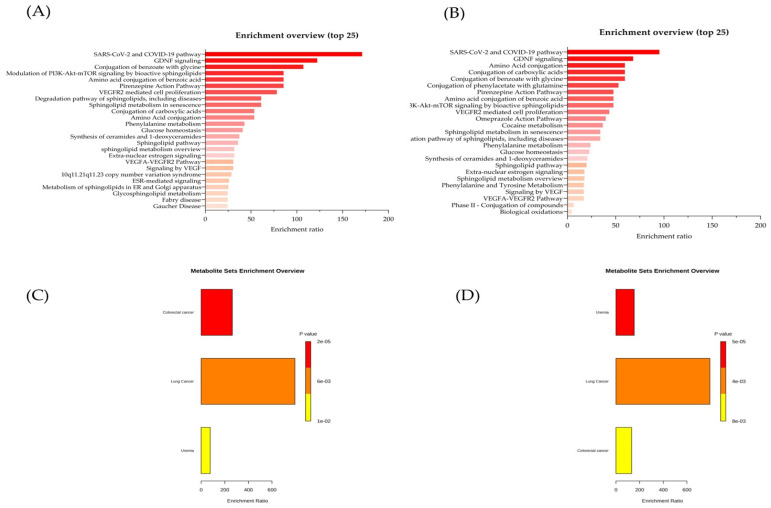
Metabolite set enrichment analysis. The list of the top 25 metabolic pathways comparing the LC group with the low-radon group (**A**) and the high-radon group (**B**) is presented. Different disease-associated metabolite sets in the LC patients were compared to the low- (**C**) and high-radon groups (**D**).

A KEGG pathway analysis was performed to explore the metabolic pathways associated with the potential metabolic markers in the LC and low- and high-radon groups. A total of 30 and 21 potential metabolites biomarkers for LC were identified within key metabolic pathways for the low- and high-radon groups, respectively. For the LC group versus the low-radon group (Figure 4A), the top five metabolic pathways were the SARS-CoV-2 and COVID-19 pathway, GDNF signaling, the conjugation of benzoate with glycine, the pirenzepine action pathway, and the amino acid conjugation of benzoic acid. In contrast, for the LC group versus the high-radon group, the top five metabolic pathways included amino acid conjugation, the conjugation of carboxylic acids, the SARS-CoV-2 and COVID-19 pathway, GDNF signaling, and the conjugation of benzoate with glycine (Figure 4B).

**Figure 5:**
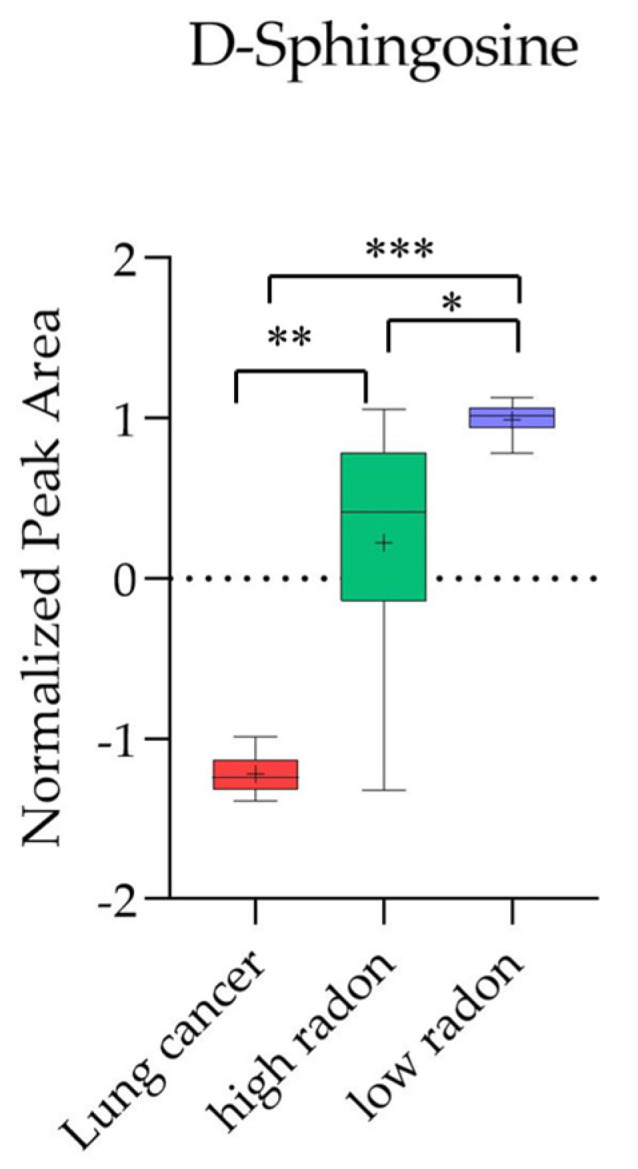
Box plots of D-sphingosine, a potential metabolic marker associated with the development of LC, are shown for the low- and high-radon groups (*, **, and *** denote *p* = 2.8 × 10^−11^, 1.0 × 10^−24^, and 8.9 × 10^−95^, respectively).

Supplemental Tables S3 and S4, further identify the hit compounds in these metabolic pathways. Notably, D-Sphingosine was identified as a major metabolite in both groups. Importantly, we conducted a KEGG pathway enrichment analysis using disease signatures from the blood samples as inputs, and this revealed that D-sphingosine played a significant role in LC development in both groups (Figure 4C,D; Table 4 and Table 5). These findings suggest that D-sphingosine may serve as a potential biomarker for LC associated with long-term exposure to indoor radon (Figure 5). As shown in Figure 5, D-sphingosine levels were significantly decreased in the LC patients compared to both the low-radon (*p* = 8.9 × 10^−95^) and high-radon (*p* = 1.0 × 10^−24^) groups. Interestingly, D-sphingosine levels were also significantly lower in the high-radon group compared to the low-radon group (*p* = 2.8 × 10^−11^), highlighting its potential as a biomarker for distinguishing between individuals similar to those in the high- and low-radon groups.

## 4. Discussion

LC is one of the leading causes of cancer-related mortality worldwide, including in Chiang Mai, affecting both males and females [1,4,6]. Major risk factors of LC in Chiang Mai include cigarette smoking, air pollution, chronic lung disease, lifestyle factors, and indoor radon exposure [5,6]. Radon is a radioactive, colorless, odorless, and tasteless gas known to be a human carcinogen [7,8,9,10,11,12,13]. Chiang Mai, particularly in areas like the Kong Khaek subdistrict, has some of the highest natural background radiation levels in Thailand [14,15,16]. Chronic low-dose indoor radon exposure in these high-background radiation areas may increase health risks, including LC. A major challenge in managing LC is late diagnosis, which significantly contributes to poor survival rates. Thus, there is an urgent need to identify noninvasive serum biomarkers for LC screening, particularly for high-risk populations with chronic low-dose radon exposure.

Metabolomics technology offers a powerful approach for discovering potential biomarkers of LC by analyzing changes in metabolic profiles [17,18]. In this study, metabolic profiling was performed using the UHPLC-HRMS technique, combined with a multivariate data analysis, ROC curve analysis, and KEGG pathway analysis. Serum samples were collected from 45 participants that were either nonsmokers or former smokers from the Kong Khaek subdistrict, and these participants were divided into the following three groups: fifteen NSCLC patients, fifteen participants in the low-radon group, and fifteen participants in the high-radon group (Table 1). All groups were matched by age and gender. The metabolic profiles of the serum samples differed among the LC patients and the participants in the low- and high-radon groups (Figure 1). The PLS-DA model effectively analyzed the differential metabolites among the three groups, as demonstrated by the observed separation between them (Figure 2B). A total of 449 metabolites were detected in the serum samples among the three groups (Figure 3A), highlighting the distinction of the LC group from both the low- and high-radon groups. Specifically, 263 and 274 differential metabolites were identified when comparing the LC group to the low- and high-radon groups, respectively (Figure 3B,C). Among these, 90 and 111 differential metabolites met the selection criteria (VIP of >1, FC of >1 or <0.5, and *p* of <0.05), clearly separating the LC group from the low- and high-radon groups (Supplemental Tables S1 and S2). This study is the first to describe and compare the metabolic markers of LC in low- and high-radon groups, providing valuable insights into potential biomarkers associated with indoor radon exposure in high-risk populations.

A ROC analysis was used to identify the potential biomarkers that significantly distinguished the LC patients from the individuals in the low- and high-radon groups (AUC of ≥0.9, 95% CI: 0.82–1.0), demonstrating high specificity and sensitivity for LC screening. Significant alterations in the serum levels of 30 and 21 metabolic markers were detected in the low- and high-radon groups, respectively, which were associated with LC development (Table 2 and Table 3). To further investigate the metabolite interactions associated with LC, a KEGG pathway enrichment analysis was performed using all the potential biomarkers listed in Table 2 and Table 3 as inputs for each group. The analysis identified D-sphingosine as a major component of sphingolipid metabolism and a key metabolite in the pathways associated with the LC patients in both the low- and high-radon groups (Figure 4A,B; Supplemental Tables S3 and S4). More importantly, according to the analysis of the metabolites associated with LC in both the low- and high-radon groups (Figure 4C,D and Figure 5; Table 2, Table 3, Table 4 and Table 5), the AUC values for D-sphingosine ranged from 0.96 to 1.00, with high specificity and sensitivity (>0.93) for evaluating LC development. D-sphingosine significantly distinguished the LC patients from both the low-radon group (*p* = 8.9 × 10^−95^) and high-radon group (*p* = 1.0 × 10^−24^), as well as between the low- and high-radon groups (*p* = 2.8 × 10^−11^). These findings suggest that lower serum levels of D-sphingosine could be utilized as a biomarker for identifying LC associated with chronic low-dose indoor radon exposure. Furthermore, decreased serum levels of D-sphingosine may also serve as a biomarker for distinguishing between individuals similar to those in the high- and low-radon groups.

Sphingolipids, including ceramide, sphingosine, and sphingosine-1-phosphate, are essential components of membrane lipids that play critical roles in carcinogenesis. Ceramide is primarily converted to sphingosine, which is subsequently converted to sphingosine-1-phosphate by sphingosine kinases. Sphingosine serves as a key regulator of various cellular processes, including tumor growth, cell proliferation, metastasis, immune response, and apoptosis [20,21,22]. Consequently, reduced sphingosine levels may correlate with an increased risk of LC development. Previous studies have shown that sphingosine levels are lower in LC patients compared to healthy controls [23,24]. This aligns with findings by Zhang et al., who reported decreased sphingosine levels in lung tumor tissue relative to noncancerous tissue [25]. Our results confirm that sphingosine is a promising candidate biomarker for screening LC in high-risk individuals residing in areas with elevated natural background radiation. As expected, residents living in high-radon areas exhibit a greater risk of developing LC compared to those in low-radon areas. This may be attributed to reduced sphingosine levels resulting from alterations in the sphingosine kinase pathway, which plays a role in LC development (Figure 5). A review of the literature suggests that decreased sphingosine levels could be linked to changes in the sphingosine kinase pathway and sphingosine-1-phosphate metabolism [26,27,28,29,30,31]. However, further investigation is needed to elucidate the mechanisms underlying these alterations and their relationship with radon exposure and LC.

This study has several limitations. First, the sample size was relatively small, and a larger sample size would be beneficial to validate these findings. Second, only NSCLC patients were included, which may limit the generalizability of the results to other types of LC. Third, all participants were either nonsmokers or former smokers, making it challenging to recruit participants, particularly LC patients. Fourth, the underlying mechanisms of the sphingosine pathway associated with LC development in the low- and high-radon groups remain unclear. Further studies with larger sample sizes and a broader participant demographic are warranted to confirm these findings and explore the mechanisms underlying the role of the sphingosine pathway in LC development.

## 5. Conclusions

To our knowledge, this is the first metabolomics-based study to investigate the effects of chronic low-dose radiation exposure on humans from high background radiation areas in Thailand. Using the UHPLC-HRMS technique, we identified distinct metabolic biomarkers of LC in the low- and high-radon groups. Among these, D-sphingosine emerged as a specific metabolite associated with LC in both groups, highlighting its potential as a metabolic marker for LC linked to chronic low-dose indoor radon exposure. Our study provides new insights into metabolic biomarkers for screening LC development in high-risk individuals with prolonged exposure to indoor radon. Further large-scale studies are needed to validate our results.

## Figures and Tables

**Table 1:** Baseline information of the study population.

Characteristics	LC (NSCLC)	HC		
		Low Radon	High Radon	Total
Age in years, mean (SD)	62 (13.3)	61.1 (11.2)	61.3 (13)	61.2 (11.9)
Gender				
Male	8	8	8	16
Female	7	7	7	14

**Table 2:** Differential metabolites in the serum samples of the LC and low-radon groups.

Metabolites	VIP	FC	*p* Value	Type	AUC	C1	C2	S1	S2
1. D-Sphingosine	2.94	0.01	8.9 × 10^−95^	Down	1	1	1	1	1
2. Benzylideneacetone	2.76	0.15	2.7 × 10^−41^	Down	1	1	1	1	1
3. Hippuric acid	2.64	0.01	7.6 × 10^−33^	Down	0.99	0.96	1	0.93	1
4. Cyprodenate	2.53	0.38	1.5 × 10^−27^	Down	0.98	0.95	1	0.98	0.98
5. Gabapentin	2.42	0.14	3.5 × 10^−23^	Down	0.98	0.96	1	0.93	0.93
6. ?-Aspartylphenylalanine	2.37	0.13	1.2 × 10^−21^	Down	0.99	0.97	1	0.93	0.93
7. 2-Aminoheptanoic acid	1.96	0.4	9.3 × 10^−13^	Down	0.94	0.88	0.98	0.87	0.89
8. 8-Azabicyclo [3.2.1]octane-3,6-diol	1.92	0.18	4.2 × 10^−12^	Down	0.91	0.83	0.97	0.82	0.91
9. Propamocarb	1.88	0.1	1.2 × 10^−11^	Down	0.92	0.86	0.97	0.96	0.82
10. Ruspolinone	1.28	0.04	1.8 × 10^−5^	Down	0.92	0.84	0.98	0.91	1
11.Triethyl phosphate	2.71	3.87	2.6 × 10^−37^	Up	0.99	0.98	1	1	0.93
12. 2-Methylnaphthalene	2.55	2.94	4.2 × 10^−28^	Up	0.99	1	1	0.93	1
13. 2,3-Octadiene-5,7-diyn-1-ol	2.51	2.53	1.9 × 10^−26^	Up	0.99	1	1	0.93	0.98
14. 2-[(5E,8E)-5,8-Tetradecadien-1-yl]cyclobutanone	2.32	2.04	3.1 × 10^−20^	Up	1	1	1	1	1
15. UNII:6S7S02945H(Ethyl menthane carboxamide)	2.25	1.64	2.6 × 10^−18^	Up	0.98	0.96	1	0.93	0.91
16. (-)-Lupinine	2.15	1.6	3.1 × 10^−16^	Up	0.97	0.94	0.99	0.93	0.91
17. Estrane	2.1	1.58	1.1 × 10^−14^	Up	0.96	0.92	0.99	0.87	0.89
18. Oleamide	2.0	1.46	9.9 × 10^−14^	Up	0.94	0.89	0.98	1	0.76
19. (2,7-Dimethyloctahydro-1H-cyclopenta[c]pyridin-4-yl)methanol	1.99	1.51	3.6 × 10^−13^	Up	0.93	0.87	0.98	0.87	0.84
20. 2-[(5Z)-5-tetradecenyl]cyclobutanone	1.97	1.47	5.9 × 10^−13^	Up	0.93	0.88	0.97	0.98	0.78
21. 4-[(1S)-1-Cyclohexyl-2-(2-piperidinyl)ethyl]cyclohexanol	1.86	3.00	2.9 × 10^−11^	Up	0.91	0.84	0.96	0.82	0.84
22. Hydrocortisone succinate	1.6	27.9	3.1 × 10^−8^	Up	1	0.99	1	1	0.98
23. Sorbitan, monododecanoate	1.35	60.7	5.4 × 10^−6^	Up	0.99	0.96	1	1	0.96
24. DB2700000 (Sulfamethoxazole N4-hydroxylamine)	1.21	20.6	6.1 × 10^−5^	Up	0.99	0.98	1	0.96	0.96
25. (6S)-2,6-Anhydro-6-[(1S)-2-isopropyl-5-methylcyclohexyl]-L-gulonic acid	1.17	37.6	9.5 × 10^−5^	Up	0.94	0.88	0.98	0.91	0.89
26. 7-O-geranyl-2-O,3-dimethylflaviolin	1.16	16.3	1.1 × 10^−4^	Up	0.94	0.88	0.97	0.84	0.89
27. Idanpramine	1.1	40	2.8 × 10^−4^	Up	0.98	0.94	1	0.96	0.93
28. Pirenzepine	1.08	45.5	3.9 × 10^−4^	Up	0.98	0.94	1	0.96	0.91
29. 4-Methoxychalcone	1.05	62.6	5.7 × 10^−4^	Up	0.90	0.83	0.95	0.93	0.78
30. Canrenone	1.01	11.9	9 × 10^−4^	Up	0.92	0.85	0.97	0.87	0.82

VIP, variable importance in the projection; FC, fold change; AUC, the area under receiver operating characteristics (ROC) curve; C1, the lower limit of a 95% confidence interval; C2, the upper limit of a 95% confidence interval; S1, specificity; S2, sensitivity.

**Table 3:** Differential metabolites in the serum samples of the LC and high-radon groups.

Metabolites	VIP	FC	*p* Value	Type	AUC	C1	C2	S1	S2
1. ?-Aspartylphenylalanine	2.30	0.12	3.4 × 10^−20^	Down	0.97	0.93	0.99	0.87	1
2. D-Sphingosine	2.45	0.04	1.0 × 10^−24^	Down	0.96	0.90	1	1	0.93
3. Hippuric acid	2.38	0.05	2.8 × 10^−22^	Down	0.95	0.90	0.99	0.93	0.91
4. Aspartame	2.13	0.11	5.3 × 10^−16^	Down	0.95	0.99	0.98	0.87	0.93
5. 1-[(9Z)-hexadecenoyl]-sn-glycero-3-phosphocholine	2.02	0.45	5.3 × 10^−14^	Down	0.91	0.84	0.96	0.78	0.87
6. L-alpha-lysophosphatidylcholine	1.95	0.45	7.7 × 10^−13^	Down	0.91	0.84	0.96	0.82	0.87
7. Pirenzepine	1.11	47.1	2.2 × 10^−4^	Up	0.98	0.94	1	1	0.91
8. Nornorcapsaicin	1.14	26.0	1.4 × 10^−4^	Up	0.96	0.92	0.99	0.87	0.89
9. Hydrocortisone succinate	1.43	23.1	9.7 × 10^−7^	Up	0.96	0.92	0.97	0.93	0.87
10. (2E)-decenoic acid	1.48	7.13	3.4 × 10^−7^	Up	0.95	0.91	0.98	0.87	0.89
11. 5-Hydroxyomeprazole	1.40	305.5	1.8 × 10^−6^	Up	0.94	0.88	0.98	0.87	0.89
12. UNII:6S7S02945H(Ethyl menthane carboxamide)	2.06	1.52	8.0 × 10^−15^	Up	0.93	0.86	0.97	0.89	0.82
13. Sorbitan, monododecanoate	1.20	51.6	6.0 × 10^−5^	Up	0.92	0.86	0.98	0.96	0.87
14. (-)-Lupinine	2.04	1.58	1.9 × 10^−14^	Up	0.92	0.86	0.97	0.93	0.84
15. 2-[(5E,8E)-5,8-Tetradecadien-1-yl]cyclobutanone	1.93	1.77	1.4 × 10^−12^	Up	0.92	0.85	0.98	0.84	0.91
16. 3-Methyl-9H-carbazol-1-ol	1.14	19.8	1.5 × 10^−5^	Up	0.91	0.85	0.97	0.84	0.84
17. Metalaxyl	1.95	4.15	6.3 × 10^−13^	Up	0.91	0.85	0.97	0.87	0.89
18. MFCD00004231(3,4-Dihydroxymandelic acid)	2.06	22.7	1.0 × 10^−14^	Up	0.91	0.85	0.96	0.80	0.87
19. Anhydroecgonine	1.34	94.1	5.8 × 10^−6^	Up	0.91	0.82	0.98	0.87	0.91
20. O-Desmethyl-cis-tramadol	1.02	530.0	8.0 × 10^−4^	Up	0.91	0.84	0.96	0.84	0.87
21. N-Phenylacetylglutamine	1.74	2.80	7.3 × 10^−10^	Up	0.90	0.83	0.95	0.87	0.87

VIP, variable importance in the projection; FC, fold change; AUC, the area under receiver operating characteristics (ROC) curve; C1, the lower limit of a 95% confidence interval; C2, the upper limit of a 95% confidence interval; S1, specificity; S2, sensitivity.

**Table 4:** Diseases associated with the metabolites in the LC and low-radon groups.

Diseases	Total	Hits	Hits Compound	*p* Value
1. Colorectal cancer	54	2	Hippuric acid and Oleamide	1.8 × 10^−5^
2. Lung cancer	9	1	D-Sphingosine	1.2 × 10^−3^
3. Uremia	92	1	Hippuric acid	0.001

**Table 5:** Diseases associated with the metabolites in the LC and high-radon groups.

Diseases	Total	Hits	Hits Compound	*p* Value
1. Uremia	92	2	Hippuric acid and N-Phenylacetylglutamine	5.5 × 10^−5^
2. Lung cancer	9	1	D-Sphingosine	0.005
3. Colorectal cancer	54	1	Hippuric acid	0.008

## Data Availability

The data presented in this study are available from the authors on reasonable request.

## Supplementary Materials

The following supporting information can be downloaded at: https://www.mdpi.com/article/10.3390/cancers16244182/s1, Table S1: Key metabolites for comparing the LC and the low-radon groups (VIP of >1, FC of >1 or <0.5, and *p* of <0.05).; Table S2: Key metabolites for comparing the LC and the high-radon groups (VIP of >1, FC of >1 or <0.5, and *p* of <0.05).; Table S3: The top 25 metabolic pathways distinguishing the LC and low-radon groups.; Table S4: The top 25 metabolic pathways distinguishing the LC and high-radon groups.
