# Supplementary material for: Serum Metabolomics Study to Screen Potential Biomarkers of Lung Cancer Risk in High Natural Background Radiation Areas of Thailand: A Pilot Study
Source: Cancers (Basel). 2024 Dec 15;16(24):4182. doi: 10.3390/cancers16244182 (PMC11674310; doi:10.3390/cancers16244182)
Supplement: Supplementary file 1 [file cancers-16-04182-s001.zip › cancers-3334913-supplementary.pdf]

Supplementary Materials for

Serum Metabolomics Study to Screen Potential Biomarkers of Lung Cancer Risk in High Natural Background Radiation Areas of Thailand: A Pilot Study

Narongchai Autsavapromporn *et al*.

*Corresponding author. Email: narongchai.a@cmu.ac.th

Supplementary Table S1. Key metabolites for comparing the LC and the low-radon groups (VIP >1, FC > 1 or < 0.5 and *P* < 0.05).

Metabolites	VIP	FC	*P* value	Type
1. Triethyl phosphate	2.71	3.87	2.6×10^{-37}	up
2. 2-Methylnaphthalene	2.55	2.93	4.2×10^{-28}	up
3. 2,3-Octadiene-5,7-diyn-1-ol	2.51	2.53	1.9×10^{-26}	up
4. 2-[(5E,8E)-5,8-Tetradecadien-1-yl]cyclobutanone	2.32	2.04	3.1×10^{-20}	up
5. UNII:6S7S02945H	2.25	1.64	2.6×10^{-18}	up
6. (-)-Lupinine	2.15	1.60	3.1×10^{-16}	up
7. Estrane	2.10	1.58	1.1×10^{-14}	up
8. Oleamide	2.02	1.46	9.9×10^{-14}	up
9. (2,7-Dimethyloctahydro-1H-cyclopenta[c]pyridin-4-yl)methanol	1.99	1.52	3.6×10^{-13}	up
10. 2-[(5Z)-5-tetradecenyl]cyclobutanone	1.97	1.47	5.9×10^{-13}	up
11. PEG Monolaurate n6	1.96	1.34	8.7×10^{-13}	up
12. Hexylbenzene	1.96	1.60	8.9×10^{-13}	up
13. 4-[(1S)-1-Cyclohexyl-2-(2-piperidinyl)ethyl]cyclohexanol	1.86	3.00	2.9×10^{-11}	up
14. (2E,4Z)-N-Isobutyl-2,4-octadecadienamide	1.85	1.80	3.9×10^{-11}	up
15. NP-021018	1.75	13.4	7.5×10^{-10}	up
16. Hydrocortisone succinate	1.60	27.9	3.1×10^{-8}	up
17. 2-(4-Methylthiazol-5-yl)ethyl butyrate	1.59	633.5	3.9×10^{-8}	up
18. UROBILIN, (-)-	1.59	5.89	4.6×10^{-8}	up
19. Furfural	1.51	1.72	2.3×10^{-7}	up
20. N-Acetyl-L-Citrulline	1.44	2.22	1.0×10^{-6}	up
21. (4S)-4-[(2E)-2-Octenoyloxy]-4-(trimethylammonio)butanoate	1.40	2.85	2.0×10^{-6}	up
22. N1-(2-Cyanoethyl)-N1-(3-pyridylmethyl)-4-methyl-1-benzenesulfonamide	1.39	47.7	2.9×10^{-6}	up
23. Sorbitan, monododecanoate	1.35	60.7	5.4×10^{-6}	up
24. 2-(Propylthio)nicotinic acid	1.35	1050	5.8×10^{-6}	up
25. Tetradecanedioic acid	1.33	141.5	7.6×10^{-6}	up
26. Anhydroecgonine	1.33	90.0	8.8×10^{-6}	up
27. 5-Hydroxyomeprazole	1.32	253.8	9.7×10^{-6}	up
28. 4-{(Z)-[(4E,7Z,16Z,19Z)-1-Hydroxy-4,7,10,13,16,19-docosahexaen-1-ylidene]amino}butanoic acid	1.32	3.47	1.0×10^{-5}	up
29. ISOFENPHOS	1.32	2739.2	1.0×10^{-5}	up
30. Stearidonic acid	1.26	3.83	2.4×10^{-5}	up
31. Chlordiazepoxide	1.26	1.37	2.4×10^{-5}	up
32. 1-(4-methoxyphenyl)-2-(propylthio)-1,4-dihydropyrido[2,3-d]pyrimidin-4-one	1.26	82.2	2.5×10^{-5}	up
33. (2E)-decenoic acid	1.25	4.92	3.1×10^{-5}	up
34. 2-[(3S)-1-(1,3-Benzodioxol-5-ylmethyl)-3-pyrrolidinyl]-5-(2-methoxyphenyl)-1,3,4-oxadiazole	1.22	257.6	4.8×10^{-5}	up

35. 3-Methylglutarylcarnitine	1.21	2.7	5.2×10^{-5}	up
36. DB2700000	1.20	20.6	6.1×10^{-5}	up
37. Nicotinuric acid	1.21	1.80	6.3×10^{-5}	up
38. O-Ethyl (4-hydroxyphenyl)carbamothioate	1.20	10.68	7.6×10^{-5}	up
39. (6S)-2,6-Anhydro-6-[(1S)-2-isopropyl-5-methylcyclohexyl]-L-gulonic acid	1.18	37.6	9.5×10^{-5}	up
40. 7-O-geranyl-2-O,3-dimethylflaviolin	1.16	16.3	1.1×10^{-4}	up
41. RH0365500	1.16	2.48	1.1×10^{-4}	up
42. 2-methyl-4-ethyl-5-propyloxazole	1.12	93.5	2.2×10^{-4}	up
43. 3-amino-2-phenyl-2H-pyrazolo[4,3-c]pyridine-4,6-diol	1.12	2.09	2.3×10^{-4}	up
44. Omeprazole	1.11	56.7	2.5×10^{-4}	up
45. Idanpramine	1.10	40.0	2.8×10^{-4}	up
46. Pantothenate	1.08	2.12	3.7×10^{-4}	up
47. Pirenzepine	1.08	45.5	3.9×10^{-4}	up
48. N-Pentacosanoylglycine	1.06	1.23	4.8×10^{-4}	up
49. (2-{[(1S,4S)-4-{3,5-Dimethyl-1-[4-(2-methyl-2-propanyl)phenyl]-1H-pyrazol-4-yl}-2-cyclopenten-1-yl]amino}-2-oxoethoxy)acetic acid	1.06	952.7	4.9×10^{-4}	up
50. cyclandelate	1.06	1.24	5.1×10^{-4}	up
51. 4-Methoxychalcone	1.05	62.6	5.7×10^{-3}	up
52. 12a-Hydroxy-3-oxo-4,6-choladien-24-oic acid	1.04	1.55	6.8×10^{-4}	up
53. 3-[2-[(E)-[3-(2-carboxyethyl)-5-[(4-ethyl-3-methyl-5-oxo-pyrrolidin-2-yl)methyl]-4-methyl-pyrrol-2-ylidene]methyl]-5-[(3-ethyl-4-methyl-5-oxo-pyrrolidin-2-yl)methyl]-4-methyl-1H-pyrrol-3-yl]propanoic acid	1.02	18.35	8.4×10^{-4}	up
54. Canrenone	1.01	11.87	9.0×10^{-4}	up
55. D-Sphingosine	2.94	0.01	8.9×10^{-95}	down
56. Benzylideneacetone	2.75	0.15	2.7×10^{-41}	down
57. Hippuric acid	2.64	0.01	7.6×10^{-33}	down
58. Cyprodenate	2.53	0.39	1.5×10^{-27}	down
59. Gabapentin	2.42	0.15	3.5×10^{-23}	down
60. ?-Aspartylphenylalanine	2.37	0.13	1.2×10^{-21}	down
61. Aspartame	2.27	0.30	7.5×10^{-19}	down
62. 2GKY1ALK5L	2.03	0.45	6.4×10^{-14}	down
63. 2-Aminoheptanoic acid	1.96	0.40	9.4×10^{-13}	down
64. 8-Azabicyclo[3.2.1]octane-3,6-diol	1.92	0.18	4.2×10^{-12}	down
65. Propamocarb	1.88	0.11	1.2×10^{-11}	down
66. ??-Glu-Gly	1.73	0.37	1.2×10^{-9}	down
67. 4-[(2R)-2-(Aminomethyl)-2-(hydroxymethyl)-5-oxo-1-pyrrolidinyl]-3-(3-pentanylamino)benzoic acid	1.68	0.27	4.4×10^{-9}	down
68. Paraxanthine	1.67	0.46	6.3×10^{-9}	down
69. MFCD00020424	1.64	0.35	1.2×10^{-8}	down
70. N-Methylhexanamide	1.64	0.27	1.2×10^{-8}	down
71. Cinnamoylglycine	1.55	0.18	9.8×10^{-8}	down
72. (2E)-2-Cyano-N-(ethylcarbamoyl)-2-(methoxyimino)acetamide	1.54	0.19	1.2×10^{-7}	down
73. 6-[(1Z)-1-Propen-1-yl]-2,3,4,5-tetrahydropyridine	1.54	0.39	1.3×10^{-7}	down
74. 2-HYDROXYHIPPURIC ACID	1.54	0.1	1.4×10^{-7}	down

75. ??-Oxo-3-pyridinebutanal	1.54	0.15	1.4×10^{-7}	down
76. 4-(allyloxy)-1,2-dihydroquinolin-2-one	1.51	0.24	2.4×10^{-7}	down
77. 3-Methylxanthine	1.50	0.44	3.3×10^{-7}	down
78. Pregabalin	1.47	0.48	5.5×10^{-7}	down
79. 2,4-Quinolinediol	1.46	0.18	6.5×10^{-7}	down
80. Pivagabine	1.45	0.40	8.5×10^{-7}	down
81. 1,7-Dimethyluric acid	1.44	0.19	1.1×10^{-6}	down
82. 2-Hydroxyquinoline	1.44	0.15	1.1×10^{-6}	down
83. Piperidione	1.42	0.42	1.5×10^{-6}	down
84. 2-Propionylthiazole	1.40	0.07	2.6×10^{-6}	down
85. 3-Methyloxindole	1.36	0.16	4.5×10^{-6}	down
86. 1-Methyl-4-[(8E)-10-methyl-6-methylene-8-undecen-2-yl]benzene	1.36	0.30	4.6×10^{-6}	down
87. Ruspolinone	1.28	0.04	1.8×10^{-5}	down
88. AG6542800	1.11	0.09	2.5×10^{-4}	down
89. MDMA Methylene homolog	1.02	0.36	8.0×10^{-4}	down
90. 1-Isopropenyl-3-isopropylbenzene	1.00	0.43	1.0×10^{-3}	down

Supplementary Table S2. Key metabolites for comparing the LC and the high radon groups (VIP >1, FC > 1 or < 0.5 and *P* < 0.05).

Metabolites	VIP	FC	*P* value	Type
1. UNII:6S7S02945H	2.06	1.52	8.0×10^{-15}	up
2. MFCD00004231	2.06	22.7	1.0×10^{-14}	up
3. (-)-Lupinine	2.04	1.57	1.9×10^{-14}	up
4. Metalaxyl	1.95	4.15	6.3×10^{-13}	up
5. NP-021018	1.95	17.5	7.8×10^{-13}	up
6. Oleamide	1.95	1.42	8.5×10^{-13}	up
7. Estrane	1.94	1.48	1.1×10^{-12}	up
8. 2-[(5E,8E)-5,8-Tetradecadien-1-yl]cyclobutanone	1.93	1.77	1.4×10^{-12}	up
9. 4-Methoxysalicylic acid	1.91	18.8	3.4×10^{-12}	up
10. (2,7-Dimethyloctahydro-1H-cyclopenta[c]pyridin-4-yl)methanol	1.87	1.49	1.1×10^{-11}	up
11. pseudouridine	1.87	1.52	1.2×10^{-11}	up
12. 2-[(5Z)-5-tetradecenyl]cyclobutanone	1.86	1.44	1.6×10^{-11}	up
13. Betaine	1.85	1.49	2.7×10^{-11}	up
14. cyclandelate	1.83	1.61	4.5×10^{-11}	up
15. Hexylbenzene	1.80	1.53	1.3×10^{-10}	up
16. N-Phenylacetylglutamine	1.74	2.80	7.3×10^{-10}	up
17. 4-[(1S)-1-Cyclohexyl-2-(2-piperidinyl)ethyl]cyclohexanol	1.72	3.02	1.3×10^{-9}	up
18. (4S)-4-[(2E)-2-Octenoyloxy]-4-(trimethylammonio)butanoate	1.67	3.67	4.5×10^{-9}	up
19. Guvacoline	1.67	1.97	4.8×10^{-9}	up
20. 3-amino-2-phenyl-2H-pyrazolo[4,3-c]pyridine-4,6-diol	1.65	2.92	6.4×10^{-9}	up
21. 2-Hydroxy-4,5',8a'-trimethyl-1'-oxo-4-vinyloctahydro-1'H-spiro[cyclopentane-1,2'-naphthalene]-5'-carboxylic acid	1.64	1.68	9.7×10^{-9}	up
22. Decanamide	1.62	1.36	1.6×10^{-8}	up
23. 4-{(Z)-[(4E,7Z,16Z,19Z)-1-Hydroxy-4,7,10,13,16,19-docosahexaen-1-ylidene]amino}butanoic acid	1.59	6.95	3.2×10^{-8}	up
24. 2-(4-Methylthiazol-5-yl)ethyl butyrate	1.59	647.9	3.3×10^{-8}	up
25. (2E,4Z)-N-Isobutyl-2,4-octadecadienamide	1.56	1.63	6.5×10^{-8}	up
26. Stearidonic acid	1.51	4.93	1.9×10^{-7}	up
27. 3-Methylglutarylcarnitine	1.51	4.66	2.0×10^{-7}	up
28. 5-Methylangelicin	1.51	19.9	2.1×10^{-7}	up
29. Piperine	1.50	15.8	2.3×10^{-7}	up
30. RH0365500	1.49	3.09	3.2×10^{-7}	up
31. (2E)-decenoic acid	1.48	7.13	3.4×10^{-7}	up
32. 2-{2-[5-(Ethoxycarbonyl)-2-morpholinoanilino]-2-oxoethoxy}acetic acid	1.48	1.53	3.8×10^{-7}	up
33. O-Ethyl (4-hydroxyphenyl)carbamothioate	1.47	20.4	4.9×10^{-7}	up

34. N-Acetylvaline	1.45	2.64	6.6×10^{-7}	up
35. LU3453000	1.45	1.49	7.4×10^{-7}	up
36. Hydrocortisone succinate	1.43	23.2	9.7×10^{-7}	up
37. N1-(2-Cyanoethyl)-N1-(3-pyridylmethyl)-4-methyl-1-benzenesulfonamide	1.43	51.7	1.1×10^{-6}	up
38. Prolylleucine	1.42	1.72	1.4×10^{-6}	up
39. 5-Hydroxyomeprazole	1.40	305.5	1.8×10^{-6}	up
40. N-Undecanoylglycine	1.40	1.28	1.8×10^{-6}	up
41. UROBILIN, (-)-	1.39	6.03	2.1×10^{-6}	up
42. 1-Dodecyl-2-pyrrolidinone	1.38	1.14	2.5×10^{-6}	up
43. N-(tert-Butoxycarbonyl)-L-leucine	1.35	1.50	4.9×10^{-6}	up
44. 2-(Propylthio)nicotinic acid	1.34	1063.9	5.4×10^{-6}	up
45. Tetradecanedioic acid	1.34	142.7	5.7×10^{-6}	up
46. Anhydroecgonine	1.34	94.1	5.8×10^{-6}	up
47. MFCD11045309	1.34	1.43	5.9×10^{-6}	up
48. Acetyl-L-carnitine	1.34	1.69	6.0×10^{-6}	up
49. 1-(4-methoxyphenyl)-2-(propylthio)-1,4-dihydropyrido[2,3-d]pyrimidin-4-one	1.33	92.7	7.0×10^{-6}	up
50. Normorphine	1.33	69.8	7.2×10^{-6}	up
51. Pantothenate	1.32	2.54	7.5×10^{-6}	up
52. ISOFENPHOS	1.31	2788.6	9.1×10^{-6}	up
53. NSC 92778	1.30	2.63	1.1×10^{-5}	up
54. 5-methylthioribose	1.30	1.41	1.3×10^{-5}	up
55. Theobromine	1.29	1.01	1.3×10^{-5}	up
56. promacyl	1.26	1.27	2.1×10^{-5}	up
57. carbapenem MM22383	1.26	9.80	2.3×10^{-5}	up
58. 5-(N,N-Dimethylcarbamimidamido)-2-oxopentanoic acid	1.25	3.36	2.9×10^{-5}	up
59. 6-Methyl[1,2,4]triazolo[4,3-b]pyridazin-8-ol	1.24	1.57	3.2×10^{-5}	up
60. Shogaol	1.24	1.36	3.4×10^{-5}	up
61. nalorphine	1.23	18.1	4.0×10^{-5}	up
62. Sorbitan, monododecanoate	1.20	51.6	6.0×10^{-5}	up
63. L-Glutamine	1.19	1.20	6.9×10^{-5}	up
64. fenoxycarb	1.19	68.3	7.1×10^{-5}	up
65. Hexadecanamide	1.16	1.28	1.0×10^{-4}	up
66. Nornorcapsaicin	1.14	26.0	1.4×10^{-4}	up
67. methyl 3,4,5-trihydroxycyclohex-1-ene-1-carboxylate	1.14	4.63	1.4×10^{-4}	up
68. 3-Methyl-9H-carbazol-1-ol	1.14	19.8	1.5×10^{-4}	up
69. 3-[2-[(E)-[3-(2-carboxyethyl)-5-[(4-ethyl-3-methyl-5-oxo-pyrrolidin-2-yl)methyl]-4-methyl-pyrrol-2-ylidene]methyl]-5-[(3-ethyl-4-methyl-5-oxo-pyrrolidin-2-yl)methyl]-4-methyl-1H-pyrrol-3-yl]propanoic acid	1.12	12.4	1.8×10^{-4}	up
70. 1-Hexadecanoylpyrrolidine	1.12	1.23	1.8×10^{-4}	up
71. (E)-doxepin	1.12	1.26	2.0×10^{-4}	up
72. Pirenzepine	1.11	47.1	2.2×10^{-4}	up
73. 2-[(3S)-1-(1,3-Benzodioxol-5-ylmethyl)-3-pyrrolidinyl]-5-(2-methoxyphenyl)-1,3,4-oxadiazole	1.10	227.0	2.3×10^{-4}	up

74. (6S)-2,6-Anhydro-6-[(1S)-2-isopropyl-5-methylcyclohexyl]-L-gulonic acid	1.10	34.8	2.4×10^{-4}	up
75. 7-O-geranyl-2-O,3-dimethylflaviolin	1.10	15.4	2.8×10^{-4}	up
76. DL-Stachydrine	1.08	2.81	3.2×10^{-4}	up
77. DL-Carnitine	1.08	1.60	3.4×10^{-4}	up
78. Propionylcarnitine	1.07	1.43	4.0×10^{-4}	up
79. 3-oxolauric acid	1.05	33.5	5.4×10^{-4}	up
80. Epitestosterone	1.04	1.87	5.9×10^{-4}	up
81. L-Pyroglutamic acid	1.04	1.16	6.0×10^{-4}	up
82. 3,6-Anhydro-1-O-stearoylhexitol	1.03	26.7	6.5×10^{-4}	up
83. Omeprazole	1.03	49.5	6.9×10^{-4}	up
84. 2-methyl-4-ethyl-5-propyloxazole	1.02	79.4	7.7×10^{-4}	up
85. O-Desmethyl-cis-tramadol	1.02	530.0	8.0×10^{-4}	up
86. Codeine	1.01	129.2	8.4×10^{-4}	up
87. Idanpramine	1.00	36.2	8.8×10^{-4}	up
88. 5-Pentyl-2(5H)-furanone	1.00	12.4	9.2×10^{-4}	up
89. N,O-DIDESMETHYLVENLAFAXINE-GLUCURONIDE	1.00	538.6	9.7×10^{-4}	up
90. D-Sphingosine	2.45	0.04	1.0×10^{-24}	down
91. Hippuric acid	2.38	0.05	2.8×10^{-22}	down
92. 1-Methyl-4-[(8E)-10-methyl-6-methylene-8-undecen-2-yl]benzene	1.78	0.06	2.0×10^{-10}	down
93. Propamocarb	1.64	0.08	7.9×10^{-9}	down
94. Aspartame	2.13	0.11	5.3×10^{-16}	down
95. 13,14-Dihydroretinol	1.67	0.12	6.3×10^{-9}	down
96. ?-Aspartylphenylalanine	2.30	0.12	3.4×10^{-20}	down
97. 1-Isopropenyl-3-isopropylbenzene	1.13	0.13	1.7×10^{-4}	down
98. 8-Azabicyclo[3.2.1]octane-3,6-diol	1.63	0.14	1.2×10^{-8}	down
99. 4-[(2R)-2-(Aminomethyl)-2-(hydroxymethyl)-5-oxo-1-pyrrolidinyl]-3-(3-pentanylamino)benzoic acid	1.40	0.31	1.9×10^{-6}	down
100. 2GKY1ALK5L	1.80	0.32	1.0×10^{-10}	down
101. N-Methylhexanamide	1.27	0.33	2.0×10^{-5}	down
102. MFCD00020424	1.61	0.35	2.1×10^{-8}	down
103. 2-Aminoheptanoic acid	1.55	0.36	8.3×10^{-8}	down
104. threonylphenylalanine	1.08	0.40	3.4×10^{-4}	down
105. Gabapentin	1.65	0.42	6.4×10^{-9}	down
106. 1-[(9Z)-hexadecenoyl]-sn-glycero-3-phosphocholine	2.02	0.45	5.3×10^{-14}	down
107. L-alpha-lysophosphatidylcholine	1.95	0.45	7.7×10^{-13}	down
108. Benzylideneacetone	1.12	0.46	1.9×10^{-4}	down
109. (2R)-3-{[(2-Aminoethoxy)(hydroxy)phosphoryl]oxy}-2-hydroxypropyl (9Z)-9-hexadecenoate	1.54	0.47	8.9×10^{-8}	down
110. DMU	1.51	0.48	1.8×10^{-7}	down
111. 1-[(8Z,11Z,14Z)-icosatrienoyl]-sn-glycero-3-phosphocholine	1.68	0.49	2.9×10^{-9}	down

Supplementary Table S3. The top 25 metabolic pathways distinguishing the LC and the low-radon groups.

Pathways	Total	Hits	Hits compound	*P* value
1. SARS-CoV-2 and COVID-19 pathway	5	1	D-Sphingosine	0.006
2. GDNF signaling	7	1	D-Sphingosine	0.008
3. Conjugation of benzoate with glycine	8	1	Hippuric acid	0.009
4. Pirenzepine Action Pathway	10	1	Pirenzepine	0.012
5. Amino acid conjugation of benzoic acid	10	1	Hippuric acid	0.012
6. Modulation of PI3K-Akt-mTOR signaling by bioactive sphingolipids	10	1	D-Sphingosine	0.012
7. VEGFR2 mediated cell proliferation	11	1	D-Sphingosine	0.013
8. Sphingolipid metabolism in senescence	14	1	D-Sphingosine	0.016
9. Degradation pathway of sphingolipids, including diseases	14	1	D-Sphingosine	0.016
10. Amino Acid conjugation	16	1	Hippuric acid	0.019
11. Conjugation of carboxylic acids	16	1	Hippuric acid	0.019
12. Phenylalanine metabolism	20	1	Aspartame	0.023
13. Glucose homeostasis	21	1	Hippuric acid	0.024
14. Synthesis of ceramides and 1-deoxyceramides	23	1	D-Sphingosine	0.027
15. Sphingolipid pathway	24	1	D-Sphingosine	0.028
16. Extra-nuclear estrogen signaling	27	1	D-Sphingosine	0.031
17. Sphingolipid metabolism overview	27	1	D-Sphingosine	0.031
18. Signaling by VEGF	28	1	D-Sphingosine	0.032
19. VEGFA-VEGFR2 Pathway	28	1	D-Sphingosine	0.032
20. 10q11.21q11.23 copy number variation syndrome	30	1	D-Sphingosine	0.035
21. ESR-mediated signaling	33	1	D-Sphingosine	0.038
22. Metabolism of sphingolipids in ER and Golgi apparatus	34	1	D-Sphingosine	0.039
23. Glycosphingolipid metabolism	35	1	D-Sphingosine	0.040
24. Fabry disease	35	1	D-Sphingosine	0.040
25. Gaucher Disease	35	1	D-Sphingosine	0.040

Supplementary Table S4. The top 25 metabolic pathways distinguishing the LC and the high radon groups.

Pathways	Total	Hits	Hits compound	*P* value
1. Amino Acid conjugation	16	2	N-Phenylacetylglutamine, Hippuric acid	4.6×10^{-4}
2. Conjugation of carboxylic acids	16	2	N-Phenylacetylglutamine, Hippuric acid	4.6×10^{-4}
3. SARS-CoV-2 and COVID-19 pathway	5	1	D-Sphingosine	0.011
4. GDNF signaling	7	1	D-Sphingosine	0.015
5. Conjugation of benzoate with glycine	8	1	Hippuric acid	0.017
6. Conjugation of phenylacetate with glutamine	9	1	N-Phenylacetylglutamine	0.019
7. Pirenzepine Action Pathway	10	1	Pirenzepine	0.021
8. Amino acid conjugation of benzoic acid	10	1	Hippuric acid	0.021
9. Modulation of PI3K-Akt-mTOR signaling by bioactive sphingolipids	10	1	D-Sphingosine	0.021
10. VEGFR2 mediated cell proliferation	11	1	D-Sphingosine	0.023
11. Biological oxidations	317	3	5-Hydroxyomeprazole, N-Phenylacetylglutamine, Hippuric acid	0.024
12. Omeprazole Action Pathway	12	1	5-Hydroxyomeprazole	0.025
13. Cocaine metabolism	13	1	Anhydroecgonine	0.027
14. Sphingolipid metabolism in senescence	14	1	D-Sphingosine	0.029
15. Degradation pathway of sphingolipids, including diseases	14	1	D-Sphingosine	0.029
16. Phase II - Conjugation of compounds	146	2	N-Phenylacetylglutamine, Hippuric acid	0.036
17. Phenylalanine metabolism	20	1	Aspartame	0.041
18. Glucose homeostasis	21	1	Hippuric acid	0.043
19. Synthesis of ceramides and 1-deoxyceramides	23	1	D-Sphingosine	0.047
20. Sphingolipid pathway	24	1	D-Sphingosine	0.049
21. Extra-nuclear estrogen signaling	27	1	D-Sphingosine	0.049
22. Sphingolipid metabolism overview	27	1	D-Sphingosine	0.049

23. Phenylalanine and Tyrosine Metabolism	28	1	N-Phenylacetylglutamine	0.049
24. Signaling by VEGF	28	1	D-Sphingosine	0.049
25. VEGFA-VEGFR2 Pathway	28	1	D-Sphingosine	0.049
